# Ultrasonography of the reticulum in 30 healthy Saanen goats

**DOI:** 10.1186/1751-0147-53-19

**Published:** 2011-03-14

**Authors:** Ueli Braun, Désirée Jacquat

**Affiliations:** 1 Department of Farm Animals, Vetsuisse Faculty, University of Zurich, Zurich, Switzerland

## Abstract

**Background:**

The reticulum plays a crucial role in the ruminant digestive tract because the primary cycle of rumen motility always starts with a reticular contraction. In contrast to cattle, there are only few results on the ultrasonographic examination of the reticulum in goats. Therefore, it was the goal of the present study, to describe the results of ultrasonography of the reticulum of 30 healthy Saanen goats.

**Methods:**

Ultrasonography was carried out on standing, non-sedated animals using a 5.0 MHz linear transducer. The shape, contour and motility of the reticulum were investigated. A nine-minute video recording of the reticulum was made for each goat and the frequency, duration and amplitude of reticular contractions were calculated as described for cattle.

**Results:**

The reticulum appeared as a crescent-shaped structure with a smooth contour located immediately adjacent to the diaphragm. 0.8 to 2.1 (1.41 ± 0.31) reticular contractions were seen per minute. In all goats, biphasic reticular contractions were observed. 90% of the goats also had monophasic reticular contractions, and two had triphasic contractions. During the nine-minute observation periods, there were 0 to 6 monophasic reticular contractions and 6 to 15 biphasic contractions per goat. The duration of the biphasic contractions was 6.56 ± 0.74 s, which was significantly longer than the monophasic contractions at 4.31 ± 0.81 s. The average interval between two reticular contractions was 45.06 ± 12.57 s.

**Conclusion:**

Ultrasonography of the reticulum in goats is a valuable tool to characterise the appearance and motility of this organ. In addition to the biphasic motility pattern seen in cattle the reticular motility of goats is characterized by monophasic reticular contractions. The results of the present study are an important contribution for better understanding of the reticular motility in goats.

## Background

Goats are not only kept as commercial livestock but are popular pets for hobby farmers. Although traumatic reticuloperitonitis is uncommon in goats because of their selective feeding habits, they are curious animals and may occasionally ingest foreign bodies [1,2]. Traumatic reticuloperitonitis has also been induced experimentally in goats [3]. Owners of pet goats or valuable breeding stock often expect a high level of veterinary care similar to that offered for cats, dogs and horses. This includes clinical examination, haematology, radiography, ultrasonography and computed tomography. The reticulum plays a crucial role in the ruminant digestive tract because the primary cycle of rumen motility always starts with a reticular contraction [4]. There are numerous studies on the ultrasonographic appearance of the reticulum in cattle, including the appearance and motility of the reticulum in healthy cows [5,6], in cows with traumatic reticuloperitonitis [6-8], vagal indigestion [9] and mechanical obstruction of the reticulum [10]. Other studies have investigated the effects of atropine, xylazine and scopolamine [11] and neostigmine [12] on reticular motility. The motility of the reticulum during rest, eating, rumination and stress in healthy cows has also been described [13]. Briefly, the normal bovine reticulum has a biphasic contraction pattern. An additional rejection contraction during rumination propels a bolus of ingesta into the oesophagus. Eating results in an increase in the number of reticular contractions, whereas stress has an inhibiting effect. Only one research group [6] has investigated the ultrasonographic appearance of the reticulum in goats. They reported that the caprine reticulum had biphasic contractions, similar to cattle. However, the goats were not ruminating at the time of examination and thus, rejection contractions were not recorded. We have observed that healthy goats, in contrast to healthy cows, may also have monophasic reticular contractions. The goal of the present study was to investigate the appearance and motility of the reticulum via ultrasonography in 30 healthy Saanen goats, and to determine whether monophasic contractions are a common occurrence.

## Methods

The study protocol was approved by the Animal Care Committe of the Canton of Zurich, Switzerland.

### Animals

Thirty clinically healthy, non-lactating female Saanen goats, which were 2.5 to 6.5 years (mean ± SD = 4.9 ± 1.10 years) old, were used. The goats originated from two farms and had been sold for slaughter. They were fed hay ad libitum and were not fasted for the examination. The goats were housed in two large pens, which were bedded with straw daily. After purchase, all of the goats were deemed healthy based on the results of a thorough clinical examination, a complete blood cell count, biochemical profile, urinalysis, and examination of rumen juice and faeces. Rumen juice was olive to brownish green and slightly viscous and had an aromatic odour. The pH was 7.5 ± 0.44 and chloride concentration was 16.5 ± 6.12 mmol/l. The manure had a normal colour and consistency. Fecal flotation indicated gastrointestinal nematodes in 27 goats, *Protostrongylus *spp. in 17 and *Fasciola hepatica *in three although none of the goats had clinical signs of parasitism such as diarrhoea or weight loss. The results of these examinations have been described in detail [14].

### Ultrasonography of the reticulum

The examinations were carried between 3 and 5 o'clock in the afternoon. Ultrasonographic examinations were carried out on standing, non-sedated animals as described previously [15] using a 5.0 MHz linear and a 5.0 MHz convex transducer with a penetration depth of 10 cm. The sternal region was clipped and contact gel was applied. The reticulum was first examined from the left side and then the right. From the left side, the examiner searched for the reticulum just dorsal to the sternum and evaluated the organ following the protocol described for cattle [5,13]. Its shape, contour and motility were assessed and the thickness of the reticular wall was determined electronically using the cursors on the monitor. The reticulum was then evaluated from the right side, after which the 5th to 9th intercostal spaces (ICS) were examined from dorsal to ventral on both sides with the transducer held parallel to the ribs to determine the location of neighbouring organs.

**Figure 1:**
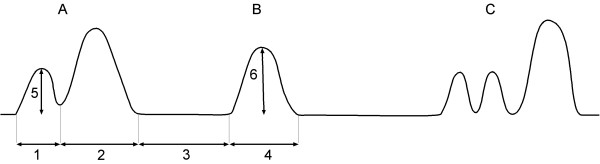
**Reticular motility**. Schematic representaion of reticular motility, modified from Seller and Stevens (1966). A Biphasic reticular contraction, B Monophasic reticular contraction, C Triphasic reticular contraction, 1 Duration of the first reticular contraction, 2 Duration of the second reticular contraction, 3 Duration of the interval between the biphasic contraction and the monophasic contraction, 4 Duration of the monophasic reticular contraction, 5 Amplitude of the first reticular contraction, 6 Amplitude of the monophasic reticular contraction.

Reticular motility was recorded during a nine-minute period using a video recorder, and analysed using the protocol established in cattle [5,13]. The number of contractions was counted in each nine-minute video recording. The length of each contraction and the time intervals between contractions were measured using a stop watch (Figure 1). The distance between the reticulum and abdominal wall was measured before and during maximum contraction for the first and second reticular contractions. An electronic ruler placed in the direction of the contraction was used to measure the amplitude of the first contraction and of monophasic contractions. The time required for the amplitude was measured and used to determine the velocity of contractions.

### Postmortem examination

After examination, the goats were slaughtered (n = 14 animals) or euthanased (n = 16 animals). A macroscopic postmortem examination of the reticulum was carried out in the slaughtered goats. The euthanased goats, which were also used in other studies [14,16,17], were frozen and cut into 1.0 to 1.5 cm-thick transverse sections. The reticulum was examined on these sections.

### Statistical analysis

The statistical software program StatView 5.1 (SAS Institute, Cary, USA) was used for analysis of the data. Frequencies, means and standard deviations were calculated. Differences were analysed using analysis of variance (ANOVA) and t-test.

## Results

### Visualisation and ultrasonographic appearance of the reticulum

The reticulum could be seen from the linea alba, the left and right paramedian regions and the 5th to 9th ICSs on both sides. The reticulum could be seen from the 6th and 7th ICSs on both sides in all the goats. From the 5th ICS, it was seen on the left in 25 goats and on the right in 15. From the 8th ICS, the reticulum was seen on the left in 22 goats and on the right in 19, and in the 9th ICS, the organ was seen on the left and right side in one goat each.

**Figure 2:**
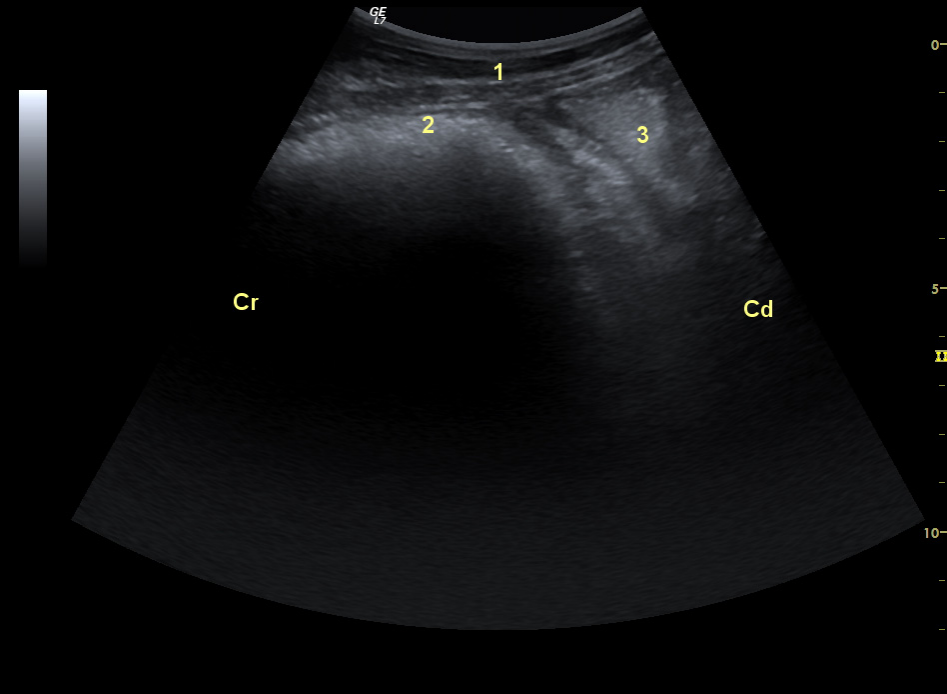
**Ultrasonogram reticulum**. Ultrasonogram of the reticulum of a three-year-old Saanen goat. Ultrasonography was carried out using a 5.0 MHz convex transducer in the left paramedian of the sternal region. 1 Abdominal wall, 2 Reticulum, 3 Abomasum, Cr Cranial, Cd Caudal.

The reticulum appeared as a crescent-shaped structure with a smooth contour and was situated immediately adjacent to the diaphragm (Figure 2). The different layers of the reticular wall could be identified in only a few goats. Similar to the bovine reticular wall [5,13], the outer tunica serosa appeared as an echogenic line, followed by the tunica muscularis as a narrow hypoechogenic band and the tela submucosa and tunica mucosa as a broad echogenic layer. In 18 goats, the mucosal folds of the reticulum were seen as irregular echogenic projections, which were a few millimeters in length and extended into the lumen. The thickness of the reticular wall ranged from 0.28 to 0.98 cm (0.54 ± 0.20 cm).

### Contractility pattern of the reticulum

Biphasic and triphasic reticular contractions were observed in the goats, similar to reticular motility in cattle [5,13]. Monophasic reticular contractions and in one goat quadraphasic contractions were seen, neither of which has been reported in healthy cattle. Biphasic contractions were similar to those in cattle with the first contraction being incomplete followed by incomplete relaxation of the reticulum. This was followed by complete contraction of the organ and then complete relaxation. In two goats that were ruminating during examination, a triphasic contraction pattern, analogous to that seen in cattle, was recorded; immediately before the biphasic contraction, a rejection contraction [13] was observed, which served to propel food into the oesophagus. Monophasic contractions consisted of a single contraction of the reticulum. In one non-ruminating goat, a quadraphasic contraction pattern was seen.

**Figure 3:**
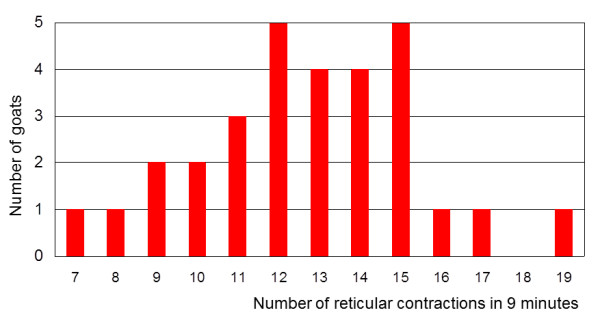
**Frequency distribution of reticular contractions**. Frequency distribution of reticular contractions during a nine-minute-observation period in 30 Saanen goats.

During the nine-minute observation period, a total of 301 (79.0%) biphasic, 77 (20.2%) monophasic, two (0.5%) triphasic and one (0.3%) quadraphasic contractions were seen in the 30 goats. This amounted to an average of 12.7 ± 2.75 contractions/9 min/goat, and the range was seven to 19 contractions (Figure 3). The number of contractions per minute varied from 0.8 to 2.1 with an average of 1.41 ± 0.31 contractions.

**Figure 4:**
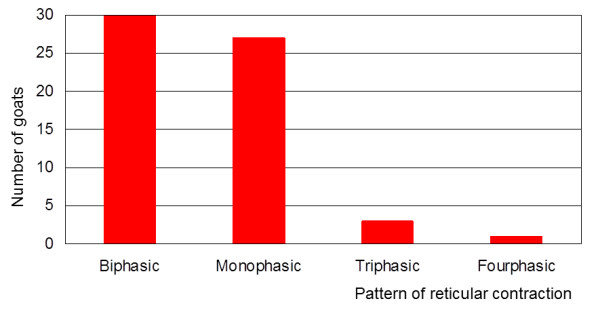
**Mode of reticular contractions**. Biphasic, monophasic, triphasic and quadraphasic reticular contractions in 30 Saanen goats.

**Figure 5:**
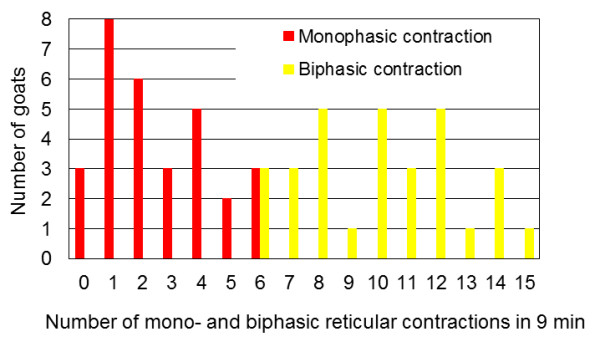
**Monophasic and biphasic contractions**. The number of monophasic and biphasic reticular contractions per goat in a nine-minute-observation period.

Biphasic reticular contractions were seen in all the goats (Figure 4). Twenty-seven (90.0%) also had monophasic contractions. In addition to monophasic and biphasic contractions, two (6.7%) goats also had triphasic contractions and one (3.3%) also had a quadraphasic contraction. The number of monophasic contractions per 9 min ranged from 0 to 6, and the number of biphasic contractions varied from 6 to 15 per goat (Figure 5). Monophasic contractions were seen irregularly between biphasic contractions in 23 goats. In the remaining four goats, monophasic and biphasic contractions alternated regularly.

### Duration of reticular contractions and interval between contractions

Biphasic contractions were a mean of 6.56 ± 0.74 s, with the first contraction being 2.77 ± 0.28 s and the second 3.88 ± 0.66) s (Table 1). Monophasic contractions were 4.31 ± 0.81 s, which was significantly shorter than biphasic contractions (P < 0.05).

**Table 1:** Variables of ruminal motility in 30 Saanen goats.

Variable	Mode of contraction	n	Mean	SD	**Min**.	**Max**.
Duration (s)	Biphasic contraction (total)	30	6.65	0.74	5.22	7.95
	
	First contraction	30	2.77	0.28	2.20	3.42
	
	Second contraction	30	3.88	0.66	2.81	5.41
	
	Monophasic contraction	26	4.31	0.81	2.87	6.00
	
	Interval between 2 contractions	30	45.06	12.57	28.94	84.50

Distance between reticulum and abdominal wall	During relaxation	30	0.04	0.21	0.00	1.14
	
	During the first contraction	30	2.15	0.77	0.79	4.71
	
	During the second contraction	11	3.34	1.35	1.86	5.86

Amplitude of reticular contractions (cm)	First contraction	30	6.97	0.92	5.10	8.65
	
	Monophasic contraction	30	5.88	1.21	3.86	8.71

Velocity of reticular contractions (cm/s)	First contraction	30	4.35	0.87	2.49	5.76
	
	Monophasic contraction	30	1.40	0.31	0.71	1.98

**Figure 6:**
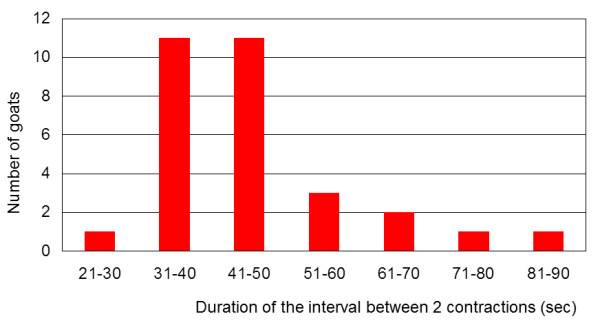
**Interval between contractions**. Duration of the interval between two reticular contractions in 30 Saanen goats.

The mean interval between two reticular contractions was 45.06 ± 12.57 s. One goat had 28.94 s between contractions, 22 goats had 31 to 50 s and 7 goats had 51 to 84.5 s (Figure 6). The time between one biphasic contraction and the following monophasic contraction was 36.1 ± 16.00 s, which was significantly shorter than the interval between a monophasic contraction and the following biphasic contraction (43.4 ± 9.10 s; P < 0.05).

### Distance between reticulum and abdominal wall during biphasic contractions

The distance between the reticulum and abdominal wall was 0.04 ± 0.21 cm during relaxation, 2.15 ± 0.77 cm during the first contraction and 3.34 ± 1.35 cm during the second contraction.

### Amplitude and velocity of reticular contractions

The first contraction had an amplitude of 6.97 ± 0.92 cm in a craniodorsal direction and a velocity of 4.35 ± 0.87 cm/s. The amplitude and velocity of the second contraction could not be measured because the reticulum moved beyond the penetration depth of the ultrasound waves and thus could not be visualised. Monophasic contractions had an amplitude of 5.88 ± 1.21 cm and a velocity of 1.40 ± 0.31 cm/s.

### Adjacent organs

The organs that were seen adjacent to the reticulum on the left side were the lungs, abomasum, rumen and liver. The lungs were seen cranial or dorsal to the reticulum from the 5th to 8th ICS in all the goats. The abomasum was adjacent and caudal to the reticulum in all the goats; it was seen from the linea alba and in the right and left paramedian regions. The rumen was immediately dorsal to the reticulum in nine goats, and the liver immediately cranial to the reticulum in one. The organs observed adjacent to the reticulum on the right side included the lungs and liver in 30 goats, the omasum in 19 and the gallbladder in two.

### Postmortem examination

Postmortem examination of the reticulum of the 30 goats revealed no abnormal findings.

## Discussion

Biphasic reticular contractions were seen in all 30 goats, monophasic contractions were also seen in 27 and triphasic contractions in two. Thus, the contraction pattern of the reticulum of goats was only partly analogous to that of cattle [4-6,13,18]. For instance, biphasic and triphasic contractions occur in both species, but monophasic contractions have not been previously reported in healthy cattle or goats. Biphasic contractions, which occurred in all the goats in our study, have also been observed in cattle [6,7,13] and goats [6]. The total number of reticular contractions in a nine-minute period ranged from 7 to 19, which corresponded to a mean of 12.7 contractions/9 min or 1.41 contractions/min. While the rate of reticular contractions in goats has not previously been reported, these results were similar to those in cattle. However, in the latter species, the contraction rate depended on whether the cow was resting (1.2 contractions/min), eating (1.6 contractions/min), ruminating (1.1 contractions/min) or stressed (1.0 contraction/min) [13]. The rate of reticular contraction in our study fell between the rates reported for eating and resting cows. The increased rate of reticular contraction in cows that are eating is thought to be due to stimulation of the buccal mechanoreceptors with subsequent stimulation of the gastric centre in the medulla oblongata and an increase in the rate [4]. It is also believed that the ingested feed stimulates the low threshold tension receptors in the reticulorumen, which are primarily responsible for the frequency, amplitude and duration of primary contractions in the reticulorumen [4,19].

One biphasic reticular contraction lasted a mean of 6.65 s in our study, which was slightly longer than the 5.50 s previously reported for goats [6], but similar to cattle in which the mean reticular contraction lasted 6.34 s [6] and 6.53 s [5]. A biphasic reticular contraction lasts 6.40 s in ruminating cows, 7.00 s in resting cows, 7.20 s in stressed cows and 7.30 s in cows that are eating [13]. The longer duration of reticular contractions in cows that are eating is thought to be attributable to the feed-filled reticulum, which results in a longer contraction time than when the organ contains less ingesta, for example at rest [13]. The short duration of contractions during rumination may be because the rejection contraction that precedes the biphasic contraction may decrease the work load of the following biphasic contraction.

The most important finding in our study was the occurrence of monophasic reticular contractions in 90.0% of the goats. To the authors' knowledge, monophasic reticular contractions have not been described in healthy cattle and goats. It has been assumed that the reticulum has biphasic contractions during non-rumination and triphasic contractions during rumination. The biphasic contraction pattern is maintained even in cattle with reticular abscesses [20]. In ruminants, monophasic reticular contractions are regarded as an exception. They have been induced experimentally in sheep after administration of a 10% copper sulfate solution [21] and were observed in two of 144 cows with vagal indigestion [9]. Interestingly, monophasic reticular contractions, as well as triphasic contractions, were not observed in a previous study that also involved Saanen goats [6]. The average monophasic reticular contraction was significantly shorter (4.31 s) than a biphasic contraction (6.65 s). The frequency of monophasic contractions was 0 to 6/9 min, which was substantially less than that of biphasic contractions (6 to 15/9 min). The duration of an individual monophasic contraction was comparable to that of the second reticular contraction of a biphasic contraction. The physiological basis of monophasic contractions in goats is speculative. They may serve to mix the reticular contents without causing the rumen to contract. Because 27 of the 30 goats had monophasic contractions and 20.2% of all reticular contractions were monophasic, it can be assumed that monophasic contractions are normal in goats.

We considered an examination time of nine minutes sufficient for adequate assessment of reticular function. In similar studies in cattle, the examination time was three minutes [5], which was later extended to nine minutes [9], although this did not yield more accurate results. In our experience repeated examinations of individual animals on subsequent days yield the same characteristic results provided that the animals are examined under the same conditions, such as during rest, eating, rumination or a stressful situation.

## Conclusions

Ultrasonography of the reticulum in goats is a valuable tool to characterise the appearance and motility of this organ. In addition to the biphasic motility pattern seen in cattle the reticular motility of goats is characterized by monophasic reticular contractions. The results of the present study are an important contribution for better understanding of the reticular motility in goats.

## Competing interests

The authors declare that they have no competing interests.

## Authors' contributions

UB initiated and planned the study and he prepared the manuscript. DJ carried out the ultrasonographic examinations under supervision of UB. Both authors have read and approved the manuscript.
